# Temporal Noise Analysis of Charge-Domain Sampling Readout Circuits for CMOS Image Sensors †

**DOI:** 10.3390/s18030707

**Published:** 2018-02-27

**Authors:** Xiaoliang Ge, Albert J. P. Theuwissen

**Affiliations:** 1 Electronic Instrumentation Laboratory, Delft University of Technology, 2628 CD Delft, The Netherlands; 2 Harvest Imaging, 3960 Bree, Belgium; albert@harvestimaging.com

**Keywords:** charge-domain sampling, CMOS image sensor, low noise, non-steady-state signal analysis, dynamic range, pixel-level amplification

## Abstract

This paper presents a temporal noise analysis of charge-domain sampling readout circuits for Complementary Metal-Oxide Semiconductor (CMOS) image sensors. In order to address the trade-off between the low input-referred noise and high dynamic range, a Gm-cell-based pixel together with a charge-domain correlated-double sampling (CDS) technique has been proposed to provide a way to efficiently embed a tunable conversion gain along the read-out path. Such readout topology, however, operates in a non-stationery large-signal behavior, and the statistical properties of its temporal noise are a function of time. Conventional noise analysis methods for CMOS image sensors are based on steady-state signal models, and therefore cannot be readily applied for Gm-cell-based pixels. In this paper, we develop analysis models for both thermal noise and flicker noise in Gm-cell-based pixels by employing the time-domain linear analysis approach and the non-stationary noise analysis theory, which help to quantitatively evaluate the temporal noise characteristic of Gm-cell-based pixels. Both models were numerically computed in MATLAB using design parameters of a prototype chip, and compared with both simulation and experimental results. The good agreement between the theoretical and measurement results verifies the effectiveness of the proposed noise analysis models.

## 1. Introduction

Advanced imaging systems for high-end applications, such as scientific and medical imaging, demand high-sensitivity CMOS image sensors (CIS). The noise performance of such CIS usually determines the ultimate detection sensitivity of the overall imaging system. However, CIS generally suffer from high temporal noise, which is typically measured by the minimum number of detectable electrons (e^−^_rms_) at the input of the pixel.

To improve the temporal noise performance of the CIS, a variety of solutions have been proposed. An effective approach is to implement a high-gain stage at the column-level, as well as to minimize the capacitance associated with the floating diffusion (FD) node at the pixel-level. By virtue of the high conversion gain (CG) along the signal path, such an approach is capable of effectively reducing the input-referred noise of each pixel. Image sensors based on this architecture have been demonstrated in serval prior designs [1,2,3,4,5,6,7,8,9,10,11], which exhibited excellent image capturing performance along with a very impressive photon-counting capability in respect of the superior noise performance. Nevertheless, the use of a fixed high-gain amplification in the pixel, either in the charge domain or the voltage domain, inevitably leads to degradation of the dynamic range (DR), thus resulting in undesired contrast loss in the final image.

To solve this problem, a charge-domain sampling pixel readout circuit based on trans-conductance (Gm)-cells has been proposed in [12,13] as an alternative to the conventional voltage-domain implementation based on source followers (SF). The linear-charging characteristic of Gm-cells enables the implementation of a variable voltage gain at the pixel level, by means of controlling the length of the charging period. As such, the CG of the overall read-out path can be programmed according to the specific application of the CIS, without the need for reconstructing or replacing the hardware. Therefore, the proposed pixel structure is able to overcome the trade-off between the low input-referred noise and high DR.

While the operational principle and implementation details of the Gm-cell-based pixel have been described in [12,13], this paper focuses on its noise characteristic. Compared to its counterpart based on source followers, a Gm-cell-based pixel operates in a non-stationary large-signal [14] manner, i.e., its bias condition changes as a function of the operating time. Therefore, the traditional temporal noise analysis method based on steady-state small-signal models does not readily apply to a Gm-call-based pixel. In view of the above issues, we propose an exact temporal noise model to guide the analysis and design of a Gm-cell-based pixel. The effectiveness of this model has been successfully verified with both simulation and prototype measurement results.

This paper is organized as follows. In Section 2, the operation principles of a Gm-cell-based pixel are briefly reviewed. Section 3 introduces the theoretical fundamentals used for the noise analysis and presents the details of the noise analysis model. Section 4 compares the analysis model with experimental results derived from simulations and measurements. Finally, a conclusion is given in Section 5.

## 2. Operating Principle and Implementation of a Gm-Cell-Based Pixel

### 2.1. Concept of Gm-Cell-Based Pixel

**Figure 1:**
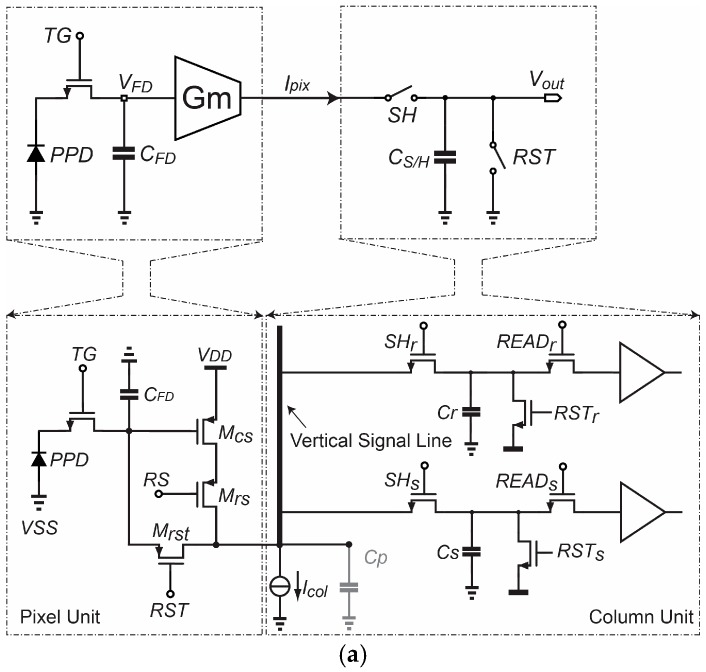

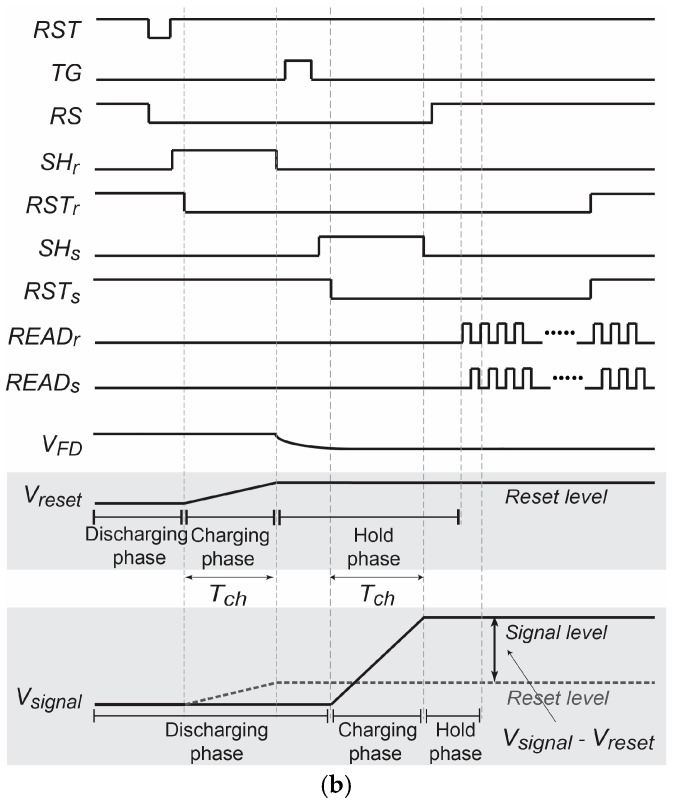
Basic schematic (**a**) and timing diagram (**b**) of the Gm-cell-based pixel with charge-domain sampling.

A simplified schematic of the Gm-cell-based pixel is shown in Figure 1a. Its basic architecture consists of a pinned-photodiode (PPD), a Gm-cell and a sample-and-hold (S/H) capacitor. Similar to [1], a single-ended cascode common-source topology has been chosen as the proposed pixel-level Gm-cell in consideration of the fill factor limitation. In addition, the column-parallel readout path has been implemented with the correlated-double sampling (CDS) S/H switches and capacitor banks for the sake of the simplicity of this proof-of-concept design.

Figure 1b illustrates the associated basic operation timing diagram. In contrast to conventional voltage-sampling pixels, the voltage on the FD node, *V_FD_*, is first converted to a signal-dependent current (*I_pix_*) by the Gm-cell, rather than buffered by a unity-gain source follower. *I_pix_* is then integrated onto the S/H capacitor (*C_S/H_*) with a programmable duration *T_ch_*, at the end of which the voltage stored on *C_S/H_* is readout as a representative of *V_FD_*. After that, *C_S/H_* is reset by the switch *RST* to clear the charge before the next sampling phase. Such sampling process essentially operates in the charge-domain, while the pixel output signal is still in the voltage domain. Its transfer function is:

(1) $$V_{out}\left(nT_{s}\right)=\frac{g_{m}}{C_{S/H}}\int_{nT_{s}}^{nT_{s}+T_{ch}}V_{FD}\left(t\right)\;dt$$

where *g_m_* is the trans-conductance of *M_CS_*, *T_S_* is the sampling period and *n* is an integer.

Equation (1) describes a boxcar sampling process [15], which can be interpreted as the convolution integral of *V_FD_* and a rectangular time window with a height of *g_m_*/*C_S/H_* and a width of *T_ch_*. Its transfer function can be written as [16]:

(2) $$H\left(f\right)=\frac{g_{m}T_{ch}}{C_{S/H}}\cdot \frac{1-e^{-j2\pi fT_{ch}}}{j2\pi fT_{ch}}$$

whose ideal magnitude transfer function can be expressed as a *sinc*-type low-pass filter with a defined DC gain:

(3) $$\left|H_{CD1}\left(f\right)\right|=\left|H_{W}\left(f\right)\right|=\frac{g_{m}T_{ch}}{C_{S/H}}\cdot \left|sinc\left(\pi fT_{ch}\right)\right|$$

Notice that the sampled voltage has been hold with zero-order hold (ZOH) process [17] after the charging phase, the ZOH model has been included as one term of the overall charge-domain sampling transfer function, which is shown below:

(4) $$\left|H_{CD1}\left(f\right)\right|=\left|H_{W}\left(f\right)\right|\cdot \left|H_{ZOH}\left(f\right)\right|=\frac{g_{m}T_{ch}}{C_{S/H}}\cdot \underset{charge-domainsampling}{\underset{︸}{\left|sinc\left(\pi fT_{ch}\right)\right|}}\cdot \underset{zero-order\;hold}{\underset{︸}{\left|sinc\left(\pi fT_{ZOH}\right)\right|}}$$

where *T_ZOH_* is the sampling period, which is equal to *T_ch_* in the case of charge-domain sampling.

**Figure 2:**
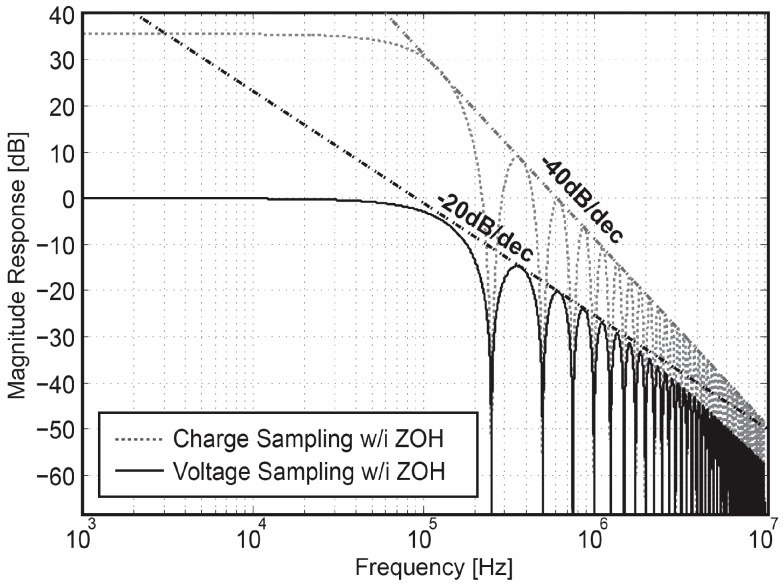
Transfer functions of the charge-domain sampling vs. voltage-domain sampling. The overall roll-off of the transfer function of charge-domain is −40 dB, one −20 dB introduced by the zero-order hold (ZOH) effect by the discrete-time sampler, the additional −20 dB introduced by the charge-domain sampler.

Intuitively, in comparison to a voltage-domain sampling readout path, a charge-domain sampling circuit introduces an additional first-order *sinc*-type low-pass filter prior to the discrete-time sampler, resulting in a twice steeper roll-off in the overall transfer function, as shown in Figure 2. The extra *sinc*-type filter features frequency notches locating at integer multiples of 1/*T_ch_*.

The above analysis reveals some important advantages of Gm-cell-based pixels for low-noise design: (1) A programmable voltage gain and −3 dB bandwidth can be realized by tuning the length of the charging window. Increasing *T_ch_* not only boosts the voltage gain, but also narrows down the readout bandwidth, both of which are beneficial for suppressing the pixel-level input-referred noise. (2) The charge-sampling process provides an additional anti-aliasing filtering, leading to further compression of the high-frequency noise components. Both features are taken into account in the following noise analysis (Section 3).

### 2.2. Periodic Filtering Model of the Charge-Domain CDS

**Figure 3:**
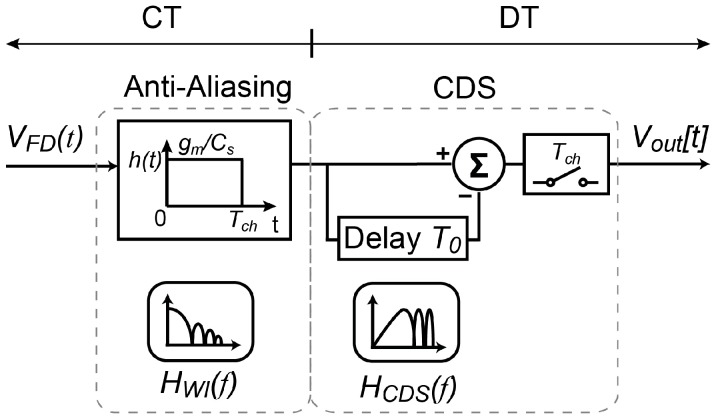
Block diagram model of the charge-domain correlated-double sampling (CDS).

CDS is widely used in CIS for low-frequency noise reduction. By subtracting the reset level sampled at *T_rst_* from the signal level sampled *T_sig_*, both the offset and the flicker noise could be effectively suppressed. The effect of the CDS noise canceller can be characterized as a discrete-time (DT) high-pass filtering operation, as analysed in [18]. The transfer function of *H_CDS_(f)* is given by

(5) $$\left|H_{CDS}\left(f\right)\right|=\left|2sin\left(\pi fT_{0}\right)\right|$$

where *T*_0_ is the sampling interval between *T_rst_* and *T_sig_*. A behavioral model of the Gm-cell-based pixel with charge-domain CDS is depicted in Figure 3. As two distinct filtering functions, namely, a continuous-time (CT) sinc low-pass filter *H_WI_*(*f*) [19] and a DT high-pass filter *H_CDS_*(*f*) are realized simultaneously, the overall transfer function of the charge-domain CDS can be written as

(6) $$\left|H_{CD2}\left(f\right)\right|=\left|H_{CD1}\left(f\right)\right|\cdot \left|H_{CDS}\left(f\right)\right|=\left|2\frac{g_{m}T_{ch}}{C_{S/H}}sinc\left(\pi fT_{ch}\right)\cdot sinc\left(\pi fT_{ZOH}\right)\cdot sin\left(\pi fT_{0}\right)\right|$$

**Figure 4:**
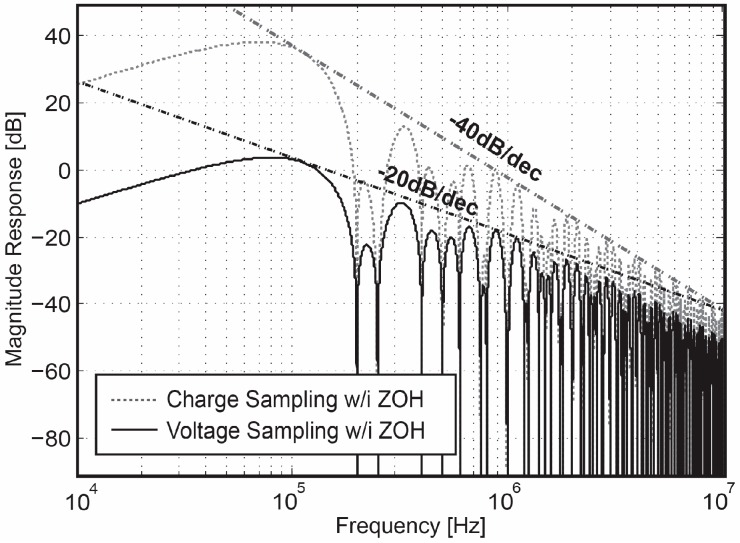
Transfer function of the charge-domain CDS vs. voltage-domain CDS.

Compared to a corresponding voltage-domain CDS transfer function, which has an equal −3 dB bandwidth, the charge-domain CDS introduces one additional group of notches. As shown by simulations in Figure 4, one group of notch frequencies is located at *k/T_ch_,* owing to the charge-sampling sinc-type filter (sinc*(πfT_ch_*)), while the other group is placed at *k/T*_0_, owing to the sinc function effect (sin*(πfT*_0_) of the CDS operation [20]. The joint effect of sinc(*πfT_ch_*) and sin(*πfT_ch_*) increases the depth of the notches thus further improving the attenuation in the stop band. As such, the charge-domain CDS not only helps in reducing the low-band noise, but also provides a greater extent of attenuation on high-frequency noise components, in comparison with the voltage-domain CDS which only features first-order low-pass filtering.

## 3. Noise Analysis of a Gm-Cell-Based Pixel

### 3.1. Nonstationary Noise Theory Analysis

**Figure 5:**
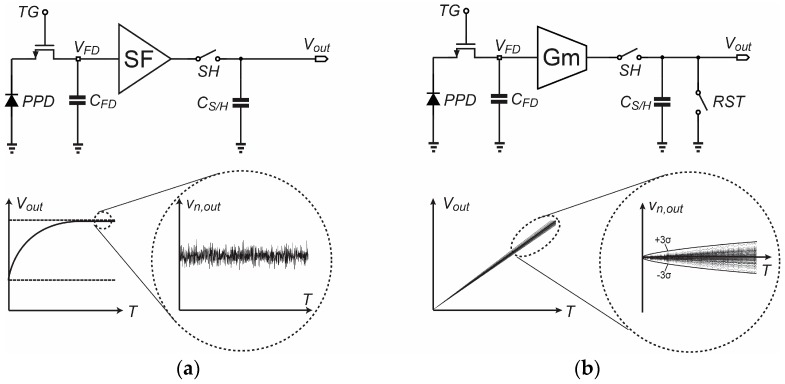
(**a**) Steady-state noise waveform for source followers (SF)-based pixel (**b**) Non-steady-state noise waveform for Gm-cell-based pixel.

Temporal noise analysis on conventional CIS readout circuits is established based on the fact that the pixel-level SF operates in the steady-state [21]. As shown in Figure 5a, in the process of the voltage-domain sampling with an exponential settling behavior, the statistics properties of the temporal noise do not vary as a function of time and can be well represented by its time-averaged root-mean-square (RMS) value. However, this prerequisite is not valid for Gm-cell-based pixels. A time-domain plot of the voltage on the S/H capacitor with superimposed random noise is conceptually shown in Figure 5b. As explained in Section II, the final output signal on the S/H capacitor is obtained through a charging process. Given the fact that the proposed read-out topology works with a large signal behavior throughout its operation, the standard deviation of the voltage distribution and hence the RMS value of the noise is no longer static with time. Therefore, the conventional noise analysis method based on steady-state models is not appropriate for Gm-cell-based pixels along with its read-out path. To quantitatively analyze the nonstationary noise, a time-domain linear analysis approach, based on the autocorrelation of nonstationary random process, has been described in [14,22]. Here, we apply a similar approach to evaluate the temporal noise characteristic of Gm-cell-based pixels.

Noise in the time-domain represents the variance of a random process, which can be derived from its autocorrelation as a function of time [23]. Suppose that the time-domain representatives of the input and output noise are *X*(*t*) and *Y*(*t*) respectively, the autocorrelation of the input noise between two time points ( $t_{1}$ and $t_{2}$ ) is *R_xx_*(*t*_1_, *t*_2_) and the time-domain impulse response of the pixel readout circuit is *h_p_*(*t*). Thus, the autocorrelation of the output noise can be derived from time-domain convolutions [14]:

(7) $$R_{YY}\left(t_{1},t_{2}\right)=h_{P}\left(t_{1}\right)*R_{XX}\left(t_{1},t_{2}\right)*h_{P}\left(t_{2}\right)$$

The variance of *Y(t)* as a function of the autocorrelation is:

(8) $$\sigma_{Y}^{2}\left(t\right)=E\left[Y\left(t\right)Y\left(t\right)\right]={R_{YY}\left(t_{1},t_{2}\right)|}_{t_{1}=t_{2}=t}$$

Equations (7) and (8) serve as the fundamental for the time-domain analysis of Gm-cell-based pixels. As can be seen, in order to investigate the output noise in the time-domain, all that is required is the input noise autocorrelation functions of different noise sources, as well as the impulse response from the pixel input voltage (*V_FD_*) to its output. During the charging phase of a Gm-cell-based pixel, both the thermal noise and flicker noise of the Gm-cell contribute to the overall output noise. In addition, the kTC noise caused by the column-level switch also affords a part of noise in the reset phase. Therefore, in the following discussions their contributions will be investigated separately.

### 3.2. Equivalent Small Signal Model and Noise Gain

**Figure 6:**
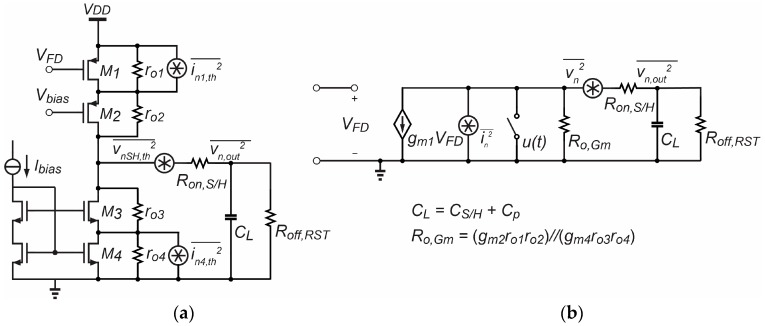
(**a**) Noise model (**b**) Small signal model of Gm-cell-based pixel.

Although a Gm–C integrator works in a large-signal behavior throughout its charging phase, its small-signal model at the completion moment of the sampling response can still be utilized for a first-order noise analysis, due to the fact that only the noise power at that point has impacts on the final decision. Figure 6 shows the noise model and the equivalent small signal model of the readout path of a Gm-cell-based pixel.

In order to facilitate the noise optimization, the mentioned output noise power needs to be referred to the FD node. For this purpose, the noise gain of the Gm-cell-based pixel must be first calculated.

According to [14], the noise gain of an integrator-like Gm-cell can be determined by the ratio of voltage slopes at the output and input ports:

(9) $$\left|A_{N}\right|=\left({\frac{dV_{out}\left(t\right)}{dt}|}_{t=T_{ch}}\right)/\left({\frac{dV_{FD}\left(t\right)}{dt}|}_{t=T_{ch}}\right)$$

The input signal V_FD_ of the Gm-cell during the charging phase can be assumed as a slow ramp and is given as:

(10) $$V_{FD}\left(t\right)=Ku\left(t\right)$$

where *K* is the input voltage magnitude. The time-domain response of a Gm-cell to a step ramp input is given by:

(11) $$V_{out}\left(t\right)=A_{0}K\left(1-e^{-t/\tau }\right)u\left(t\right)\;\mathrm{where}\;A_{0}=g_{m1}R_{OUT}=g_{m1}\left(R_{o,Gm}||\left(R_{off,RST}+R_{on,S/H}\right)\right)\mathrm{and}\tau =R_{OUT}\cdot \left(C_{S/H}+C_{p}\right)$$

where *A*_0_ *= g_m_*_1_*R_OUT_* is the DC gain of the Gm-cell at the steady-state, *τ* is the time constant of the Gm–C integrator, *R_o,Gm_* is the output impedance of the Gm-cell, *R_on,S/H_* is the on-resistance of switch *SH,* the value of which is much smaller than *R*_o,*G*_, *R_off,RST_* is the off-resistance of switch *RST* (*RSTr* and *RSTs*). Thus, the final noise gain of the Gm-cell can be described by the following expression:

(12) $${\left|A_{N}\right||}_{t=T_{ch}}=A_{0}\left(1-e^{-T_{ch}/\tau }\right)$$

Note that this result can be simplified into two special cases:

|AN|={(13a)gm1ROUT, for Tch≫τ(13b)gm1Tch/CL, for Tch≪τ

**Figure 7:**
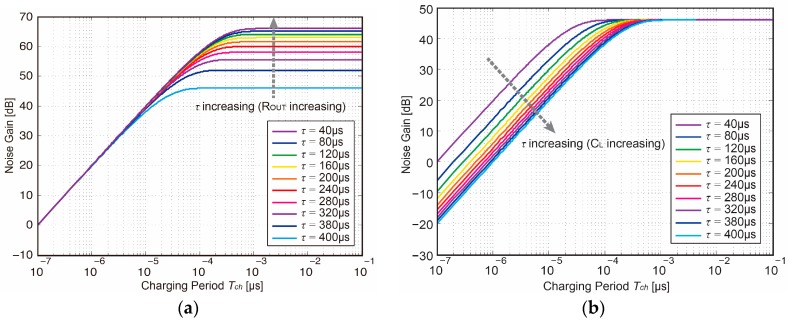
Noise gain factor as a function of charging period *T_ch_* (**a**) with *τ* and *R_OUT_* increasing (**b**) with *τ* and *C_L_* increasing.

To be more precise, a plot showing the variation of the noise gain factor as a function of charging period *T_ch_* with a wide range of time constant *τ*. As revealed in Figure 7a and Equation (13a), with a constant *T_ch_* and the time-boundary *T_ch_ >> τ*, the noise gain increases as *τ* and *R_OUT_* increasing, which showing the steady-state noise gain characteristic of a broadband amplifier. Figure 7b and Equation (13b) shows an integrator-like noise gain with the time-boundary *T_ch_ >> τ* resulting from the charge-sampling process as explained in [2], which is inversely proportional to *τ* and *C_L_* with a constant *T_ch_*.

### 3.3. Noise Model of Charging Phase

#### 3.3.1. Thermal Noise

In a Gm-cell small signal model, the impulse response from the noise current source to the output voltage *V_out_*, which is given by [14]:

(14) $$h_{P}\left(t\right)=\frac{1}{C_{L}}e^{-t/\tau }u\left(t\right)=\frac{1}{C_{S/H}+C_{p}}e^{-t/\tau }u\left(t\right)$$

where *C_S/H_* is the loading S/H capacitance, *C_p_* is the parasitic capacitance of the column net, *R_o,Gm_* is the output impedance of the Gm-cell, *τ* is the time constant of this Gm–C integrator and *u(t)* is the noise current unit step input.

Consider a white noise unit step input *u_n_*(*t*), the autocorrelation function of the thermal noise source is a Dirac delta function with an amplitude equal to its double-sided power spectral density (PSD) [23]:

(15) $$R_{XX,th}\left(t_{1},t_{2}\right)=\frac{S_{th,n}}{2}\delta \left(t_{2}-t_{1}\right)$$

where *S_th,n_* is the equivalent single-sided temporal noise PSD. According to Figure 6, the noise sources include the equivalent current noise source *i_n_* from the pixel-level Gm-cell and the equivalent voltage noise source *v_n_* from the column-level sample-and-hold switch *SH* (*SHr* and *SHs*). Thus, *S_th,n_* can be modelled as:

(16) $$S_{th,n}=4kTg_{n}+\frac{4kTR_{on,S/H}}{{R_{OUT}}^{2}}\approx \frac{8}{3}kT\left(g_{m1}+g_{m4}\right)$$

where *g_n_* = 2(*g_m_*_1_ + *g_m_*_4_)/3 is the equivalent noise trans-conductance of *i_n_*, *k* = 1.3807 × 10^−23^ J/K is the Boltzmann constant and *T* is absolute temperature in Kelvin.

By substituting Equations (14) and (15) into Equations (7) and (8), we obtain the variance of the output voltage due to the time-variant thermal noise, as given by:

(17) $$\sigma_{Y,th}^{2}\left(t\right)=\frac{S_{th,n}}{2}{\int_{0}^{t}\left|h\left(\alpha \right)\right|}^{2}d\alpha$$

with the aid of Equation (16), the above integral can be solved as:

(18) $$\sigma_{Y,th}^{2}\left(t\right)=\frac{2}{3}\cdot \frac{kT}{C_{S/H}+C_{p}}\left(g_{m1}+g_{m4}\right)R_{OUT}\left(1-e^{-2t/\tau }\right)u\left(t\right)$$

In a charge sampling circuit, only the noise at the instant of the sampling completion (*T_ch_*) has impact on the final readout noise. Accordingly, the concerned output thermal noise power of a Gm-cell-based pixel can be evaluated as:

(19) $$\bar{v_{n,th}^{2}}={\sigma_{Y,th}^{2}\left(t\right)|}_{t=T_{ch}}=\frac{2}{3}\cdot \frac{kT}{C_{S/H}+C_{p}}\left(g_{m1}+g_{m4}\right)R_{OUT}\left(1-e^{-2T_{ch}/\tau }\right)$$

On the basis of Equations (12) and (19), the input thermal noise power can be derived by:

(20) $$\bar{v_{in,th}^{2}}={{\sigma_{Y,th}^{2}\left(t\right)|}_{t=T_{ch}}/\left|A_{N}|\right|}_{t=T_{ch}}^{2}=\frac{2}{3}\cdot \frac{kT}{\tau }\cdot \left(\frac{1}{g_{m1}}+\frac{g_{mz}}{g_{m1}^{2}}\right)\cdot coth\left(\frac{T_{ch}}{2\tau }\right)$$

={(21a)4kT⋅23(1gm1+gm4gm12)⋅14τ, for Tch≫2τ(21b)4kT⋅23(1gm1+gm4gm12)⋅12Tch, for Tch≪2τ

**Figure 8:**
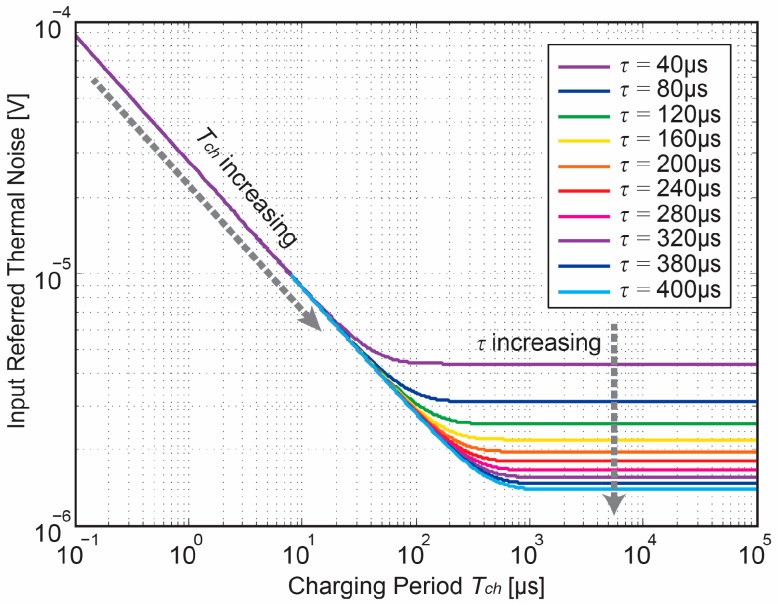
Input referred thermal noise as a function of charging period *T_ch_*.

As Equation (20) contains a hyperbolic function of the ratio of the charging time *T_ch_* and time constant *τ*, the time limits *T_ch_ >> τ* and *T_ch_* << *τ* are thus of interest. Figure 8 showing the variation of the input thermal noise power with a wide range of time constant *τ*. With a fixed *T_ch_*, the input-referred thermal noise decreases as *τ* increases at the time-boundary of *T_ch_* << *τ*. Its noise behavior is identical with the input-referred thermal noise power in common single-pole steady-state systems. On the other hand, if *T_ch_* << *τ*, the input-referred thermal noise linearly reduces as *T_ch_* gets longer for a given *τ*. Within this region, the thermal noise becomes *T_ch_*-dependent and behaves as an integrator-like noise. As such, this interesting characteristic offers an orientation to the thermal noise estimation in the specific design of Gm-cell-based pixels.

#### 3.3.2. Flicker Noise

Flicker noise in CIS refers to those noise sources whose PSD is inversely proportional to the frequency. The flicker noise PSD sourced from the input MOS transistor of the Gm-cell can be modeled as:

(22) $$S_{1/f,n}=\frac{K}{C_{ox}\cdot A}\cdot \frac{1}{f}\cdot {\left(g_{m1}+g_{m4}\right)}^{2}$$

where *K* is a process-dependent constant, *C_ox_* is the unit oxide capacitance of the MOS gate, and *A* is the channel area. In contrast to thermal noise, the time-domain response of flicker noise is a nonstationary process [22].

(23) $$R_{XX,1/f}\left(t_{1},t_{2}\right)=h_{1/f}\left(t_{1}\right)*R_{XX,th}\left(t_{1},t_{2}\right)*h_{1/f}\left(t_{2}\right)$$

where $h_{1/f}\left(t\right)$ is the impulse response of an ideal 1/f noise-shaping filter:

(24) $$h_{1/f}\left(t\right)={\left(\frac{2f_{C}}{t}\right)}^{1/2}u(t)$$

Here, *f_c_* is the corner frequency of the flicker noise, which is relevant to the process and transistor parameter:

(25) $$f_{C}=\frac{K}{C_{ox}\cdot A\cdot S_{th,n}}\cdot {\left(g_{m1}+g_{m4}\right)}^{2}$$

Based on Equations (23)–(25), the autocorrelation of flicker noise can be expressed as:

(26) $$R_{XX,1/f}\left(t_{1},t_{2}\right)=\frac{K{\left(g_{m1}+g_{m4}\right)}^{2}}{C_{ox}A}\int_{0}^{\infty }\frac{1}{{\left[\mu \cdot (t_{2}-t_{1}+\mu )\right]}^{1/2}}u(\mu )d\mu$$

Equation (26) appears as a divergent integral function of time [24,25,26,27] and does not have a finite limit. To address this issue, characterization of the flicker noise is often reasonably limited to a finite length of observation time window [24] (or a limited bandwidth in the frequency domain [23]). The minimum of this time window (*t_min_*) is defined by the reciprocal of the upper limit of the concerned frequency range, i.e., the flicker corner frequency (*f_c_*), while the total operation time of the readout circuit (*t_op_*) determines the maximum. Based on this approximation, the autocorrelation of flicker noise can be written as [23]:

(27) $$R_{XX,1/f}\left(t_{op},t_{1},t_{2}\right)≅\frac{K{\left(g_{m1}+g_{m4}\right)}^{2}}{C_{ox}A}\ln\frac{4t_{op}}{\left|t_{2}-t_{1}\right|},\;\mathrm{where}\;\frac{1}{f_{C}}\ll \left|t_{2}-t_{1}\right|\ll t_{op}$$

By substituting Equations (14) and (27) into Equations (6) and (7), the variance of the pixel output voltage owing to flicker noise can be expressed as:

(28) $$\sigma_{Y,1/f}^{2}\left(t\right)=\frac{{\left(g_{m1}+g_{m4}\right)}^{2}R_{OUT}K}{2\left(C_{S/H}+C_{p}\right)C_{ox}A}\int_{0}^{t}\left(\ln\frac{4t_{op}}{\alpha }\right)\cdot \left[1-e^{-2(t+\alpha )/\tau }\right]u(\alpha )d\alpha$$

Similarly, the output flicker noise at the sampling instant is evaluated at *T_ch_*:

(29) $$\bar{v_{n,1/f}^{2}}={\sigma_{Y,1/f}^{2}\left(t\right)|}_{t=T_{ch}}$$

However, the integral in Equation (28) does not have an analytic solution. Therefore, Equations (28) and (29) must be numerically computed in MATLAB to get a quantitative evaluation of the flicker noise power. Note that *t_op_* should be assigned with a sufficiently large value to ensure the accuracy of approximation (typically around one hour [22]). Take the CDS effect into consideration, the impulse response of the ideal 1/f noise-shaping filter are assumed as *h_1/f_*(*T_ch_*) and *h*_1*/f*_(*T*_0_ + 2*T_ch_*). Therefore, the autocorrelation of the flicker noise with CDS is given as:

(30) $$R_{XX,1/f}\left(T_{ch},T_{0}+2T_{ch}\right)=h_{1/f}\left(T_{ch}\right)*R_{XX,th}\left(T_{ch},T_{0}+2T_{ch}\right)*h_{1/f}\left(T_{0}+2T_{ch}\right)$$

where *T*_0_ is the interval period between two samples (reset level and signal level) which is assumed as *T*_0_ *= T_ch_* + 1 µs. Consequently, the output flicker noise power after CDS can be defined by:

(31) $$\sigma_{Y,1/f}^{2}\left(t\right)=\frac{{\left(g_{m1}+g_{m4}\right)}^{2}R_{OUT}K}{2\left(C_{S/H}+C_{p}\right)C_{ox}A}\int_{T_{ch}}^{2T_{ch}+T_{0}}\left(\ln\frac{4t_{op}}{\alpha }\right)\cdot \left[1-e^{-2(2T_{ch}+T_{0}+\alpha )/\tau }\right]u(\alpha )d\alpha$$

**Figure 9:**
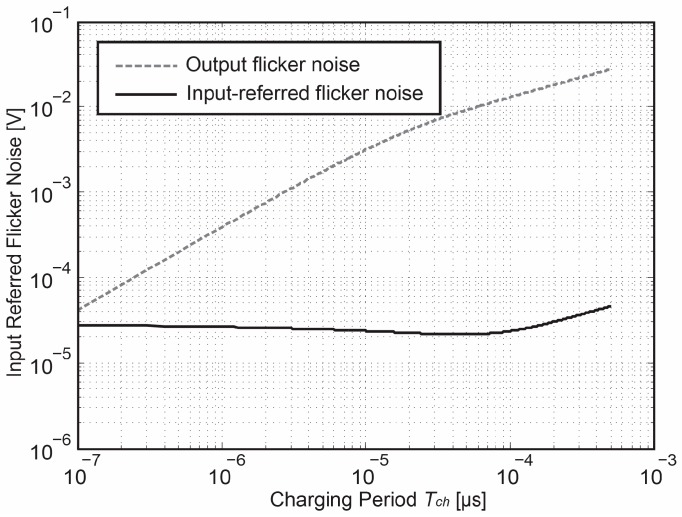
Input referred flicker noise as a function of charging period *T_ch_*.

As a brief proof, Figure 9 numerically plot the flicker noise output power as a function of charging time *T_ch_*. In contrast to thermal noise output power whose value reaches steady-state until *T_ch_* ≈ 2*τ*, flicker noise is continuously accumulated with an increasing *T_ch_*, which agrees with the theoretical analysis of the flicker noise in frequency domain.

According to our circuit level simulations, the corner frequency f_c_ is around 500 kHz, which is higher than the equivalent noise bandwidth of the proposed circuit, and thus the flicker noise obviously appears even beyond the noise bandwidth. As a result, the input-referred flicker noise is highly dependent on *T_ch_* and it is effectively reduced through increasing *T_ch_*. On the contrary, as the Gm-cell enters into the steady-state region when *T_ch_* gets longer. The input-referred flicker begins to increase due to a constant noise gain and noise bandwidth.

### 3.4. Noise Model of Discharging Phase

**Figure 10:**
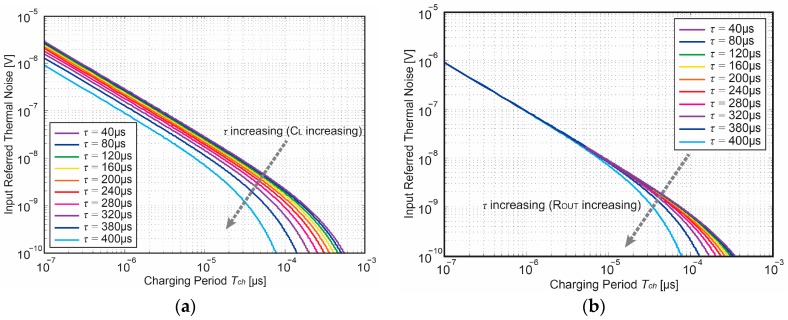
Input referred kTC noise as a function of charging period *T_ch_* during discharging phase (**a**) with *τ* and *C_L_* increasing (**b**) with *τ* and *R_OUT_* increasing.

In order to segregate the sampling operations between two adjacent frames, the S/H capacitor is discharged by switching on *RST* (*RSTr* and *RSTs*) before the next new frame. As the switch operation during this process is considered to have reached stationary levels with an exponential settling behavior, the noise is therefore exhibit as the steady-state. By using the first-order low-pass filter transfer function, the thermal noise power caused by switch *RST* is calculated as:

(32) $$\bar{v_{n1,kTC}^{2}}=\int_{0}^{\infty }4kTR_{on,RST}\frac{1}{1+{\left(2\pi fR_{on,RST}C_{L}\right)}^{2}}=\frac{kT}{C_{L}}$$

where *R_on,RST_* is the on-resistance of switch *RST*. Different from the voltage-domain sampling circuit, the charging phase follows the switch off of *RST*. As a consequence, part of the noise charge on *C_L_* is discharged in the charging phase with a non-stationary random process and thus the resulting noise power from *RST* is given as:

(33) $$\bar{v_{n,kTC}^{2}}={\sigma_{Y,kTC}^{2}\left(t\right)|}_{t=T_{ch}}=\frac{kT}{C_{L}}-{\sigma_{n1,kTC}^{2}\left(t\right)|}_{t=T_{ch}}=\frac{kT}{C_{L}}-\frac{kT}{C_{L}}{\int_{0}^{t}\left|h\left(\alpha \right)\right|}^{2}d\alpha =\frac{kT}{C_{L}}e^{-2T_{ch}/\tau }$$

where the term $e^{-2T_{ch}/\tau }$ represent the amplitude degrading during the charging phase. The value of the kTC noise from the discharging phase are also numerically investigated, with results presented in Figure 10a,b.

### 3.5. Overall Input-Referred Noise

Consequently, the overall input-referred temporal noise power of a Gm-cell-based pixel can be calculated by:

(34) $$\begin{array}{ll} \bar{v_{n,in}^{2}} & =\frac{{{2\sigma_{Y,th}^{2}\left(t\right)|}_{t=T_{ch}}+\sigma_{Y,1/f}^{2}\left(t\right)|}_{t=T_{ch}}+{2\sigma_{Y,kTC}^{2}\left(t\right)|}_{t=T_{ch}}}{{\left|A_{N}\right|}^{2}}\\ & =\frac{{{2\sigma_{Y,th}^{2}\left(t\right)|}_{t=T_{ch}}+\sigma_{Y,1/f}^{2}\left(t\right)|}_{t=T_{ch}}+{2\sigma_{Y,kTC}^{2}\left(t\right)|}_{t=T_{ch}}}{{\left[g_{m1}T_{ch}/\left(C_{S/H}+C_{p}\right)\right]}^{2}},where\;T_{ch}\ll \tau \end{array}$$

The combination of formulas (20), (31) and (33) provides an effective way to predict and calculate the temporal noise power of Gm-cell-based pixels in the time domain. Given the fact that the proposed circuit operates as a Gm–C integrator, *T_ch_* should be settled with the range of *T_ch_ << τ*. Applying the device parameters used for the design of the CIS chip as listed in Table 1, the noise components of the readout circuits and the resulting total noise are calculated in MATLAB, which is shown in Section 4 as a comparison of measurement result.

## 4. Implementation and Experimental Results

The test sensor with the proposed pixel architecture has been fabricated in a 0.18 µm 1P4M standard CIS process technology. The test pixels have been divided into six sub-groups, each of which includes 20 (H) × 32 (V) pixels and features the same pixel pitch of 11 µm. For flexibility, the digital logic, which implements the charging clocks *T_ch_* and other operating clocks are realized off-chip. By performing these double charging processes, the resulting voltage level *V_reset_* and *V_signal_* are held on *C_r_* and *C_s_* respectively and are sequentially readout from the CIS chip via multiplexers and output buffers. An off-chip 16-bit ADC with an LSB of 30 µV has been implemented on the PCB to convert the analog output voltage levels into digital signal. The voltage subtraction of the reset level and the signal level (*V_reset_* − *V_signal_*) is then performed in the digital domain with the aid of an NI-IMAQ (National Instruments–Vision Acquisition Software) 16.2. In this way, we realize the CDS in digital domain and obtain the period-controlled amplified video signal *V_signal_* − *V_reset_* with the charge-domain CDS.

**Figure 11:**
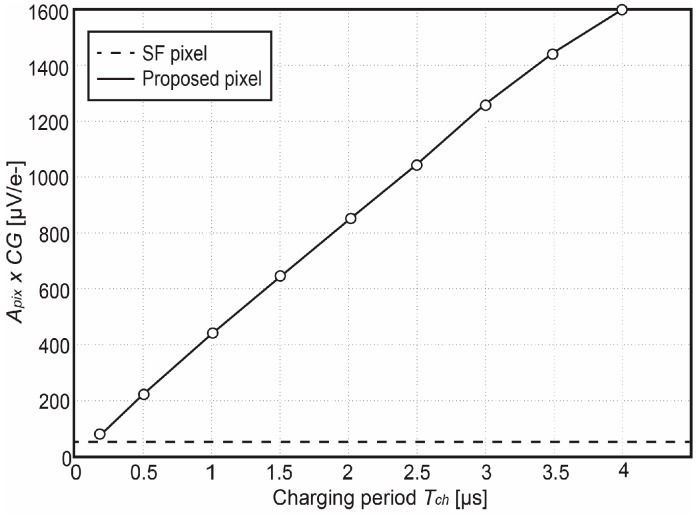
Measured conversion gain (*CG_FD_* × *A_pix_*) as a function of the charging period *T_ch_*. [12].

A critical parameter for the evaluation of the temporal noise is the conversion gain. The pixel-level conversion gain *CG_tot_* associated with the period-controlled function has been measured by using the photon transfer curve (PTC) measurement technique. Figure 11 shows the measured conversion gain *CG_tot_ = CG_FD_* × *A_pix_* of the fabricated Gm-cell-based pixel, where *CG_FD_* is the conversion gain at the FD node. To separately investigate the gain factor *A_pix_* of the charge-sampling pixel, we also measured the *CG_FD_* of a unity-gain pMOS SF-based 4T-pixel [28] as a reference for comparison, whose the FD node is laid out with the same area as the proposed pixel. Note that the *CG_FD_* of the SF-based pixel is measured as 55 μV/e^−^, which indicates that the nominal value *A_pix_* of the charge-sampling pixel is around ×30. The measurement results show that *CG_tot_* can be programmable from 50 µV/e^−^ to 1.6 mV/e^−^ when a charging period from 100 ns to 4 µs is applied.

**Figure 12:**
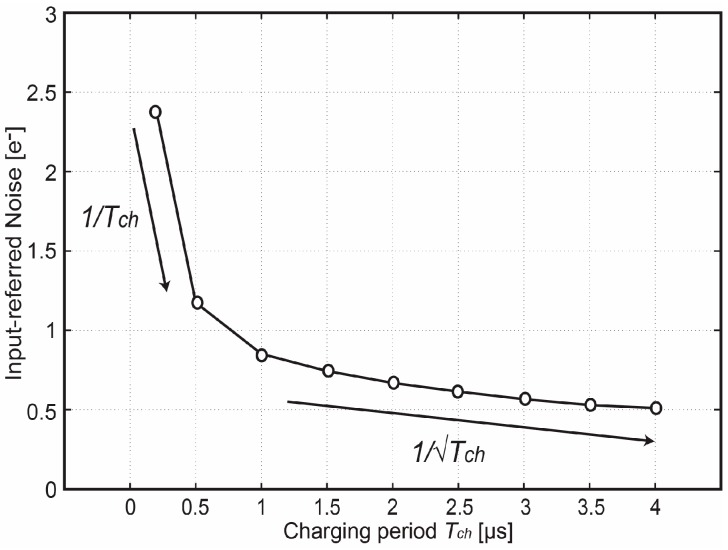
Measured input-referred noise as a function of the charging period *T_ch_* [12].

**Figure 13:**
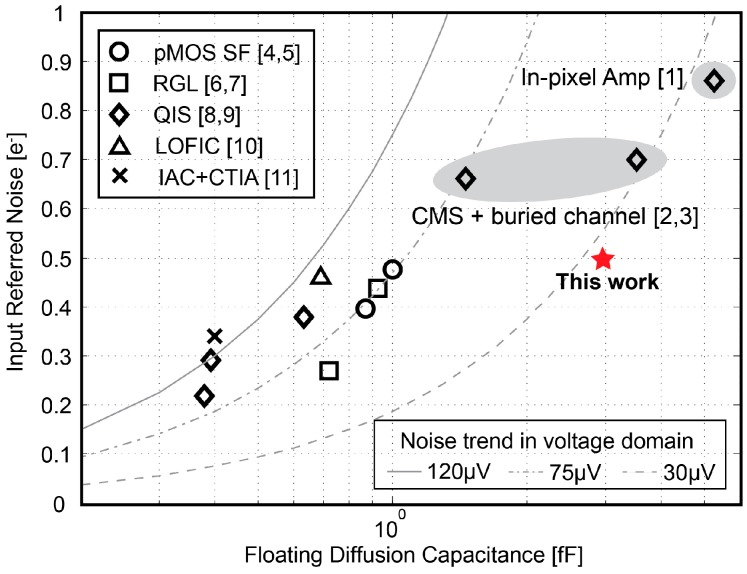
Comparison of input-referred noise in the electron domain vs. FD capacitance, and noise trend in the voltage domain with reported image sensors. The values are based on the best guess with the known values of *CG_FD_* in reported publications [13].

The temporal noise characterization has been done in the dark and implemented by keeping the transfer gate TG off during the measurement period. Figure 12 shows the measured input-referred noise of the proposed pixel as a function of *T_ch_*. The noise-reduction tendency initially is proportional to 1/*T_ch_* and later becomes proportional to 1/√*T_ch_*. This result indicates that the Gm-cell-based pixel not only reduces the noise originating from the exceeding circuits connected at the back of the pixel as a result of the signal amplification of the charge-sampling technique, but also suppresses the thermal noise generated by the pixel level circuit as a result of noise-bandwidth reduction. At *T_ch_* = 4 µs, the pixel achieves an input-referred noise of 0.51 e^−^_rms_. In addition, when referred the noise back to the input of the signal chain in the voltage domain by dividing its corresponding gain factor *A_pix_*, the lowest measured input-referred noise level is found around 27 µV, which is shown and compared with other state-of-the-art low-noise CIS in Figure 13. This figure presents that an improvement in figure-of-merit regarding the read-out noise reduction was successfully obtained by using the proposed Gm-cell-based pixel and charge-domain CDS technique.

**Figure 14:**
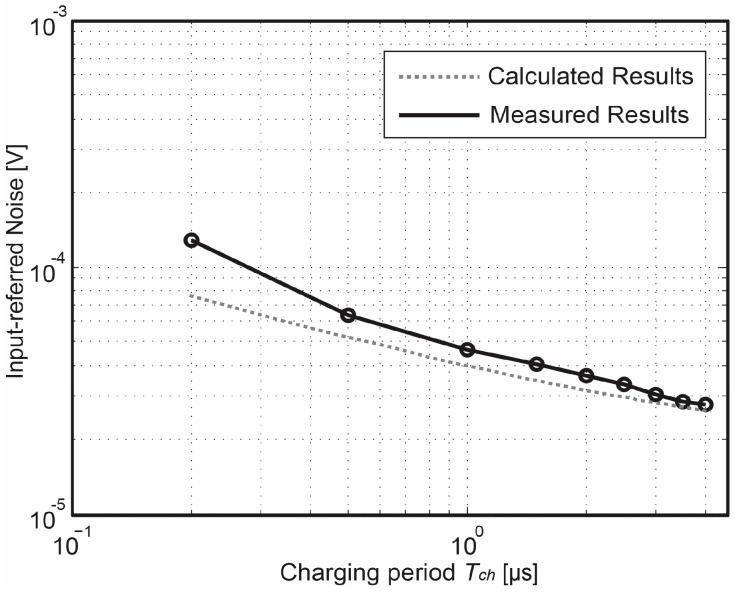
Input-referred noise in voltage domain as a function of *T_ch_* for measured and simulated results.

For verification of the time-domain noise analysis model, Figure 14 shows the measured input-referred noise with a comparison to the simulation results in voltage domain. In the calculation results described above, noise due to the clock jitter effect and sample and hold process, as well as the noise generated from the board-level succeeding readout circuits are ignored. As a result, there is a noise value deviation between the calculation and measurement results. Moreover, because of the trans-conductance is *V_FD_*-dependent and the Gm-cell is open loop, the *g_m_* variation degrades the pixel output linearity, leading to a noise reduction factor deviation between two results. As Figure 14 indicates, the noise reduction tendency obtained from the calculation model shows a steeper slope than the measurement results. However, the measured and calculated results show a reasonable agreement on the noise reduction tendency, demonstrating the validity of the noise calculation by using the time-domain noise analysis model.

## 5. Conclusions

A Gm-cell-based CMOS image sensor pixel structure that realizes a tunable conversion gain with a charge-domain CDS scheme was proposed for applications in low-noise high-DR image sensors. In contrast to conventional CIS pixel architectures, a Gm-cell-based pixel operates in a large-signal manner, and its noise behaves as a function of time. To allow a precise and predictive noise performance optimization for such type of pixels and their readout circuits, a non-stationery thermal and flicker noise analysis model based on a time-domain approach is presented and discussed in this paper. By comparing the numerical results derived from the proposed models with both simulation and experimental results, which showed a reasonable agreement, the effectiveness of the theoretical analysis model was verified.

## Figures and Tables

**Table 1:** Device parameter used for the noise estimation.

Parameter	Value	Parameter	Value
*g_m_*_1_	30 µS	A	3 µm (W) × 0.5 µm (L)
*C_p_*	2 pF	K	1 × 10^−25^
*R_o,Gm_*	20 MΩ	Cox	4.3 fF/µm^2^
*k*	1.38 × 10^−23^	fc	500 kHz
*T*	300 K	top	~1 h
